# Surgical Treatment of Solitary Metachronous Adrenal Metastasis from Urothelial Carcinoma of the Urinary Bladder

**DOI:** 10.17925/EE.2023.19.1.94

**Published:** 2023-01-13

**Authors:** Dimitrios Politis, Panagiota Konstantakou, Konstantinos Bramis, Krystallenia I Alexandraki, Ariadni Spyroglou, George Mastorakos, Ioannis Anastasiou, Ioannis Papaconstantinou, Meletios A Dimopoulos

**Affiliations:** 1. Second Department of Surgery, Aretaieion University Hospital, Medical School, National and Kapodistrian University of Athens, Greece; 2. Endocrinology Department, Aretaieion Hospital, Medical School, National and Kapodistrian University of Athens, Greece; 3. First Department of Urology, National and Kapodistrian University of Athens, Laikon University Hospital, Athens, Greece; 4. Department of Clinical Therapeutics, National and Kapodistrian University of Athens, Alexandra Hospital, Athens, Greece

**Keywords:** Adrenal metastasis, urothelial carcinoma, metastatic urothelial carcinoma, metastatic urothelial bladder carcinoma, adrenalectomy, surgical treatment of adrenal metastasis

## Abstract

Urothelial cancer is a common neoplasm and metastatic disease correlates with a poor prognosis. Isolated adrenal gland metastases of urothelial carcinoma are quite rare, and management options can decide a patient’s prognosis. Herein we report the case of a 76-year-old man with a metachronous solitary adrenal metastasis from a bladder carcinoma, who underwent adrenalectomy as part of his treatment. Furthermore, we discuss the cases of solitary adrenal metastases of urothelial carcinoma available in the literature, to identify key features to direct appropriate treatment of this rare metastatic site of urothelial cancer and improve prognosis and survival. Still, further prospective studies are needed to design effective therapeutic strategies.

Bladder cancer (BC) is the second most commonly diagnosed urological neoplasm worldwide.^1^ Approximately 10–15% of patients already have metastases in lymph nodes, lungs, liver and bones at diagnosis.^2,3^ Metastatic BC has a poor prognosis, with a 5-year overall survival (OS) rate of 10% and median OS of only 15 months.^4–6^ The vast majority (>90%) of BCs are urothelial carcinomas (UCs). Around 70% of these newly diagnosed UCs are categorized as superficial disease, with the remaining 30% representing muscle-invasive disease.^7^ The latter, after treatment with radical cystectomy, recurs locally and/or distally in 35% of patients, resulting in a 5-year recurrencefree survival of 58–68%.^8^ Over two-thirds of patients with UC recurrence die within the following 12 months.^8^ Developing visceral metastases is a well-recognized poor prognostic factor.^9^ Specifically, adrenal metastases occur in 14% of patients with BC, with solitary adrenal metastasis being very rare.^10^

The adrenal glands are the fourth most common metastatic site for all cancers after the lung, liver and bone.^11,12^ Synchronous, bilateral adrenal metastases are rare (<0.5%),^11–14^ except with lymphomas, where the prevalence of bilateral adrenal involvement reaches 71%.^15,16^ The abundant sinusoidal blood supply of the adrenal glands and the possible communication between the pulmonary and retroperitoneal lymphatic pathways have been postulated to facilitate the metastatic process.^12^ However, such a supply is present in the spleen, which is seldom a site of metastasis.^17^ Metastases occur usually at the border between the adrenal cortex and medulla.^18^ In most cases, imaging can differentiate benign from malignant adrenal lesions.^11,19–22^ In the context of known previous malignancy, presence of an adrenal lesion is highly suspicious of metastatic disease, with diagnostic tools – adrenal biopsy and immunohistochemistry for highly sensitive and specific markers of adrenocortical origin, such as steroidogenic factor 1 – distinguishing metastasis from primary adrenocortical carcinoma.^11,21,23,24^

In a study of 464 patients with metastatic adrenal lesions, only 4% were symptomatic.^25^ Clinical presentation included lower chest, back or abdominal pain, a palpable abdominal mass, or symptoms and signs related to adrenal insufficiency and adrenal haemorrhage.^26,27^ According to previous reports, <1% of the cases of adrenal insufficiency (Addison’s disease) occur due to metastases in the adrenal gland, since sparing 10% of the adrenal gland is sufficient for maintaining adequate adrenal function.^28,29^ Nevertheless, evaluating adrenal function in patients with metastases is always warranted to exclude adrenal insufficiency.

According to current guidelines, systemic platinum-based chemotherapy is the standard of care for metastatic UC, yet with a limited 5-year OS of 13–17%.^30^ Immunotherapy with immune checkpoint inhibitors (ICIs) is a new second-line treatment option, with a reported overall response rate of 15–21% and durable response in a subset of patients.^31^ On this ground, metastasis-directed therapy with surgical resection could be considered in patients with UC recurrence. The benefit of this approach has been shown in other cancer types, such as colorectal and renal cancer with similar proliferation index as UC, where metastasectomy is a reasonable practice, improving survival.^32^ Regarding metastatic UC, although limited, available data seem quite promising.

We describe herein a patient with UC of the bladder who, after initial resection of the primary tumour and adjuvant chemotherapy, had disease recurrence in the right adrenal gland. Adrenalectomy was performed and, despite disease progression, the patient exhibits satisfactory performance status and longer than expected OS.

## Methods

Written informed consent was obtained from the patient for the publication of the case report and the accompanying images. A literature search of the PubMed database using the terms “metastatic urothelial bladder carcinoma”, “metastatic urothelial bladder cancer”, “metastatic urothelial carcinoma/cancer and adrenal metastases”, “urothelial carcinoma/cancer and metastasectomy”, “metastatic urothelial carcinoma/cancer and surgical resection of metastases”, “surgical resection of adrenal metastases” was performed and the identified reported cases are commented upon herein in relation to the present case.

## Case presentation

**Figure 1:**
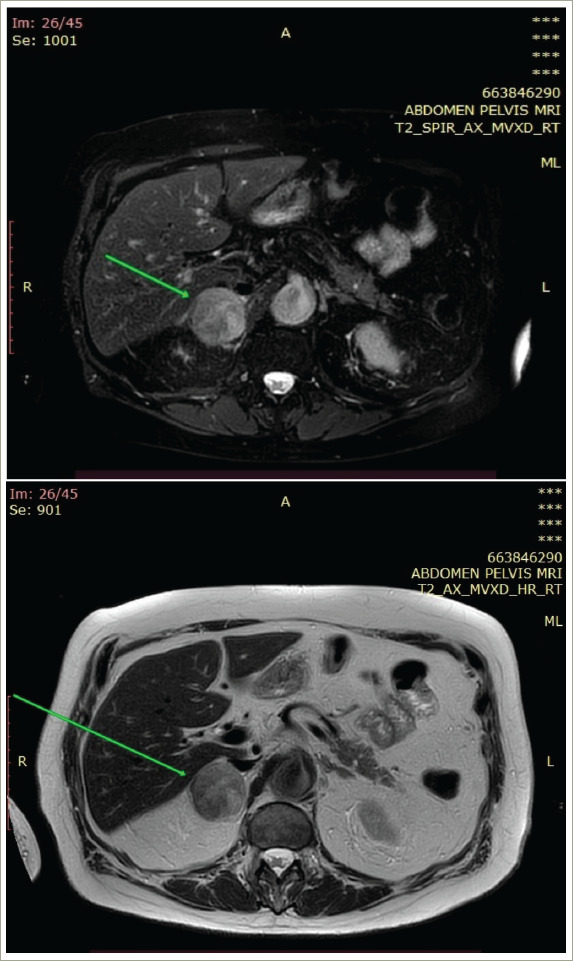
Magnetic resonance imaging depicting the right adrenal mass (arrow)

In September 2021, a 76-year-old man with history of urothelial BC resected and treated with adjuvant chemotherapy and immunotherapy was referred to our department for further evaluation and management of a suspicious adrenal mass. The patient underwent transurethral tumour resection (TUR) followed by radical cystectomy and prostatectomy immediately after initial diagnosis in March 2019. The pathology report documented urothelial BC with Tumour-Node-Metastasis status pT0N3Mx (the cystectomy specimen revealed no residual tumour since a TUR preceded). Subsequently he received three cycles of adjuvant chemotherapy with gemcitabine and carboplatin from June 2019 to November 2019. One year after operation, at followup magnetic resonance imaging, a mass with a maximum diameter of 3.6 cm was detected in the right adrenal gland (*Figure 1*). At that point, immunotherapy with nivolumab, a programmed death-1 inhibitor, was initiated. Radiation therapy was considered but was not employed, since the adrenal tumour progressed in size. However, due to the patient’s poor response to immunotherapy after 1 year of treatment, he was switched to vinflunine and treated for 7 months.

A subsequent computed tomography (CT) scan, performed in August 2021, revealed a notably larger adrenal metastasis, reaching 5 cm × 6 cm × 6.5 cm, with no other metastatic foci. The patient remained asymptomatic. In October 2021, right adrenalectomy was performed and the patient had an uneventful recovery. The pathology report described extensive infiltration of the adrenal gland from a highgrade UC, with peri-focal necrosis and inflammation confirming UC metastasis. Immunohistochemical staining was CK903+, p63+, inhibin-, GATA3+, K20+/-and K7-. A follow-up 18F-fluorodeoxyglucose positron emission tomography/CT at 5 months revealed peritoneal infiltration, and palliative chemotherapy with docetaxel was initiated and continues to this day. The patient, having been operated on for solitary adrenal metastasis and treated with chemotherapy and immunotherapy regimens, is alive 3 years and 3 months after initial diagnosis and 8 months after right adrenalectomy.

## Discussion

We present a rare case of a patient with UC that had a metachronous, solitary, adrenal metastatic focus with a relatively favourable clinical course after unilateral adrenalectomy. This case confirms the feasibility of metastasectomy in the management of recurrent UC disease and, most importantly, its contribution to achieving disease control and prolonged progression-free survival (PFS).

Metastatic UC of bladder, ureter or renal pelvis is a highly aggressive disease with limited therapeutic options. Current guidelines recommend combined chemotherapy as first-and second-line treatment but median OS is only 15 months.^33^ Platinum-based chemotherapy is the mainstay of treatment but complete response and cure is still elusive despite high initial response rates and the recent introduction of ICIs into the treatment of this cancer.^30,31^ To date, there is no second-line standard of care universally accepted for platinum-refractory metastatic UC.^34^ In patients progressing after platinum-based combination chemotherapy for metastatic disease, guidelines suggest entry into a clinical trial or, alternatively, single-agent therapy (paclitaxel, docetaxel or vinflunine where available).^35^ Accordingly, in our patient, immunotherapy was initiated after disease progression with platinum-based chemotherapy, with further stepwise appropriate changes of the treatment plan (vinflunine, docetaxel) based on overall response. Therapy with docetaxel is still on-going, and our patient maintains a relatively good performance status and quality of life.

**Table 1:** Reports of a solitary adrenal metastasis of bladder urothelial carcinoma

Publication	Wyler S, 2005^50^	Honda M, 2012^51^	Washino S, 2012^49^	Present case
Age, years	63	64	69	76
Sex	Male	Male	Male	Male
Operation	Radical cystectomy	Radical cystectomy	Radical cystoprostatectomy	Radical cystoprostatectomy
Stage	Grade 3, pT1N0, Ki-67 index 50%	Grade 3, pT3aN1M0	pT2bN0	pT0N3Mx
Histology	Transitional cell carcinoma	UC	High-grade UC	High-grade UC
Metastasis characteristics	Right adrenal mass 3.7 cm × 4 cm	Left adrenal mass	Left adrenal mass 5 cm × 4 cm	Right adrenal mass 3.6 cm maximum diameter; increase to 5 cm × 6 cm × 6.5 cm within 8 months
Time to metastasis detection	Synchronous	3 years	8 months	1 year
Surgical treatment	NR	Left adrenalectomy	Left adrenalectomy	Right adrenalectomy
Chemotherapy	NR	Post-cystectomy chemotherapy	Post-left adrenalectomy, three cycles of gemcitabine, carboplatin, dexamethasone	Post-cystectomy chemotherapy three cycles; immunotherapy, chemotherapy 7 months
Follow-up	2 years post-operative increase in size 6.4 cm × 6.3 cm, nonfunctional right adrenalectomy	NR	Recurrence after 6 months in right adrenal gland; three cycles of methotrexate, vinblastine, epirubicin, cisplatin. No response; right adrenalectomy	Recurrence after 5 months with peritoneal metastases; palliative chemotherapy
PFS (post-adrenalectomy)	2 years	6 years	3 years	Follow-up still on-going

*NR = not reported; PFS = progression-free survival; UC = urothelial carcinoma.*

Current preclinical evidence supports targeting the vascular endothelial growth factor (VEGF) pathway as a treatment option.^36^ However, clinical trials documented conflicting data on anti-VEGF efficacy.^37,38^ According to a recent meta-analysis assessing optimal second-line therapy for metastatic UC, an OS advantage of immunotherapy over taxanes but not over vinflunine was found. Moreover, adding an anti-VEGF receptor agent to chemotherapy did not significantly benefit OS or PFS.^39^

To improve outcome, surgical resection of metastases has been proposed by several investigators. Surgical resection of oligometastatic disease is an established practice in the management of patients diagnosed with other solid tumours, but data on metastasectomy in UC are scarce.^40,41^ The European Association of Urology (EAU) guidelines suggest considering surgery along with radiotherapy and/or chemotherapy in the context of an individualized approach for patients with local recurrence and distant oligometastatic disease.^35^

Published data so far, which included only a retrospective uncontrolled case series and two meta-analyses with considerable limitations, suggest a possible benefit from surgical resection of UC recurrence in selected patients, mostly when in combination with systemic chemotherapy.^42,43^ A recent multicentre retrospective analysis that included 326 patients with metastatic UC reported improved survival in patients who received standard chemotherapy and underwent surgery compared with patients treated with chemotherapy only, provided that disease was limited to one metastatic site.^43^ In line with this observation, a recent retrospective, single-institutional case series of 22 patients who underwent metastasectomy with oligometastatic UC – including one patient with an adrenal metastatic focus – reported a 5-year OS rate of 51.4%, with significantly better outcomes in patients with small (<8 mm) or solitary pulmonary lesions.^44^ Previous retrospective studies with a limited number of patients with metastatic UC undergoing metastasectomy (combined in most cases with systemic chemotherapy) have demonstrated a survival advantage with favourable results on 5-year OS rate (28–31%) and median OS after surgery from 18 to 27 months.^45–48^

In the absence of prospective, randomized controlled trials, strict eligibility criteria for metastasectomy cannot be clearly determined. Most centres and experts advocate it in patients with good response to chemotherapy, recurrence at site of initial surgery, presence of solitary metastasis, feasibility of complete resection, and no evidence of rapid disease progression.^44,45,48^ In our case, adrenalectomy was decided on grounds of evident solitary metastasis, the patient’s good performance status, institutional expertise and feasibility of complete tumour resection. Indeed, operation was uncomplicated and recovery uneventful.

Isolated adrenal metastases in UC are rare and there is little reported evidence on surgical resection of adrenal metastases in UC.^49^ Washino et al. published an interesting case of metastatic UC with isolated, asynchronous adrenal metastasis to bilateral adrenal glands in a 69-year-old man after radical cystoprostatectomy and adjuvant chemotherapy.^49^ Left adrenalectomy, and subsequent right adrenalectomy after new recurrence in the right adrenal gland 6 months after left adrenalectomy, were performed. The patient had an uneventful recovery on steroid replacement therapy and remained free of disease 3 years later.^49^ In addition, a 63-year-old patient with bladder UC and a growing solitary adrenal metastasis (detected prior to cystectomy) had right adrenalectomy 2 years after radical cystectomy; he had a favourable outcome, with no evidence of disease at 2-year follow-up.^50^ Another case has been reported of isolated adrenal metastasis originating from bladder UC, 3 years after radical cystectomy, treated with left adrenalectomy and chemotherapy, and with remission at 6-year follow-up.^51^ Patient characteristics are summarized in *Table 1*.^49–51^

With regard to complications and safety of metastasectomy, a population-based analysis of outcomes in older adults with metastatic UC reported a complication rate at first metastasectomy of 10% within 30 days of discharge, with highest risk noted when the procedure site was the liver (16%) and the lung (15%).^48^ Additionally, the 30-day mortality rate after metastasectomy was 10%, which is comparable to the post-operative mortality rate of primary radical cystectomy in patients >65 years, suggesting a generally acceptable safety profile in this population.^48,52^ In line with the published data mentioned above, our patient had an uncomplicated adrenalectomy with quick recovery despite his advanced age.

## Conclusions

In summary, as supported by our presented case, in appropriately selected patients with UC with solitary adrenal metastasis, adrenalectomy constitutes a safe and effective approach that, when combined with systemic therapy, may result in disease control and improve survival.
